# Different Analytical Modes on Automatic Hematology Analyzers for Testing Quality Control Materials and Commutability Assessment

**DOI:** 10.1111/ijlh.70179

**Published:** 2026-07-22

**Authors:** Bingshuang Ren, Ya Cheng, Hongping Ba, Weijia Cheng, Xuwei Zhou, Hui Zhen, Shimin Wu, Jie Gong

**Affiliations:** ^1^ Department of Clinical Laboratory Wuhan Center for Clinical Laboratory Wuhan China; ^2^ Center for Clinical Laboratory General Hospital of the Yangtze River Shipping Wuhan Brain Hospital Wuhan China

**Keywords:** automatic hematology analyzers, commutability, complete blood count, quality control, quality control materials

## Abstract

**Introduction:**

Hematological disorders, critical for diagnosis, and monitoring, rely on accurate complete blood count results from automated analyzers, the reliability of which depends on robust quality control (QC). However, the ability of commutability of commercial hematology QC materials (HQCM) to behave like patient samples across different systems, and the impact of analytical modes on results are not fully characterized. This study aimed to evaluate the analytical performance and commutability of two HQCM brands (Bioyuan and XC) across two hematology analyzers (Sysmex XN‐1000 and Mindray BC‐6800 Plus).

**Methods:**

We systematically compared results between QC and sample measurement modes, between manual and autoloader sampling modes, and assessed commutability using clinical samples as a reference.

**Results:**

The differences between QC and sample modes were analyzer and parameter specific, with WBC, HCT, and MCV most affected on the Sysmex system. Manual versus autoloader sampling on the Mindray analyzer introduced significant differences for most parameters across concentrations. Commutability assessment revealed that neither HQCM was universally commutable for all four directly measured parameters (WBC, RBC, HGB, PLT); noncommutability was frequent, particularly, at extreme concentrations, indicating significant matrix effects.

**Conclusion:**

This study highlights that analytical (sampling mode and measurement mode) variables, along with material‐dependent matrix effects, can substantially influence QC results and their interpretation. Laboratories must standardize protocols and critically select QC materials to ensure reliable hematology testing and effective quality assurance. Future work should focus on developing more commutable reference materials and validating these findings in multicenter clinical settings.

## 1 Introduction

The complete blood count (CBC) is one of the most frequently requested tests in laboratory medicine, measuring key parameters, such as white blood cells (WBC), red blood cells (RBC), hemoglobin concentration (HGB), and platelets (PLT), and is applied in a wide range of clinical situations [1, 2, 3]. International quality control (QC) is crucial for obtaining reliable results, and its effectiveness depends on factors such as the performance of control materials and the hematology analyzer, as well as the parameter settings for different analytical modes [4, 5, 6]. However, in current clinical practice, systematic investigation into the impact of different testing modes and their effects on QC materials is lacking.

According to the International Standards Organization (ISO) 15189, laboratories should use independent third‐party control materials, either instead of or in addition to any control materials supplied by the reagent or instrument manufacturers [7]. Commercial hematology and third‐party control materials are widely used in international QC practices and External Quality Assessment (EQA) because of their relatively long shelf life [8, 9, 10, 11]. Ideally, the control materials should exhibit characteristics identical to those of homogeneous specimens within the testing system [12, 13]. However, in practice, this aspect is often overlooked. Specifically, for both international QC and EQA, manufacturers of control materials and analyzers recommend using a dedicated “control mode” for analyzing these materials, whereas the “sample mode” is used for patient samples. This oversight ignores the potential quality risks that arise from noncommutable control materials. The acquisition of commutable commercial QC materials poses considerable challenges because of their high cost and limited shelf life [10], thereby restricting their widespread use in most laboratories and in large‐scale EQA programs. Consequently, many medical laboratories and EQA providers opt for more economical alternatives; however, commutability has not yet been evaluated.

The objectives of this study were first to determine whether statistically significant differences existed between the measurements performed in QC mode and the sample mode for the two Hematology QC materials (HQCM) brands on both analyzers. Second, we evaluated the differences between the manual (open vial) and autoloader (closed vial) sampling modes on one analyzer to understand the preanalytical impact on QC measurements. Third, we assessed the commutability of commercially available HQCM between the two analyzer platforms. By investigating these interrelated factors, this study aimed to provide evidence‐based insights that can guide laboratories in optimizing their QC protocols, selecting appropriate control materials and ultimately enhancing the reliability and harmonization of hematological testing, which are crucial for accurate patient management of a wide array of hematological disorders.

## 2 Materials and Methods

### 2.1 Quality Control Materials for Hematology

HQCM from two different manufacturers, each available at high, medium, and low concentrations, was used. The HQCM from Bioyuan (Lot: 2534, Bioyuan Biotechnology Co., Zhengzhou, China) was human‐derived and had an unopened shelf life of 3 months and a postopening stability of 15 days. The HQCM from Xin Jiankangcheng (XC) (Lot: 0525, Xin Jiankangcheng Biotechnology Co., Chengdu, China) was animal‐derived and had an unopened shelf life of 6 months, although its in‐use stability was 7 days after opening. The high, medium, and low concentration levels of the two HQCM are listed in Tables S1 and S2, respectively.

In addition to the QC materials, 26 clinical whole blood samples were obtained from the General Hospital of the Yangtze River Shipping. The use of anonymised residual samples for method comparison was approved by the General Hospital of the Yangtze River Shipping Ethics Committee (Ethics Approval No.: YL20250062) and the Wuhan Centre for Clinical Laboratory (WHCCL) Ethics Committee (Ethics Approval No: KY2025001), and was conducted in accordance with the ethical principles of the Declaration of Helsinki.

### 2.2 Instruments

Two automatic hematology analyzers were used: XN‐1000 (Sysmex Co., Kobe, Japan) and BC‐6800 Plus (Mindray, Shenzhen Mindray Bio‐Medical Electronics Co., Shenzhen, China) which are the two most prevalent brands in clinical laboratories throughout this region. Both analyzers provide a five‐part WBC differential along with nearly 30 additional reportable parameters [14, 15]. The detection principles of the two hematology analyzers were identical. RBC and platelets were counted and sized using direct‐current impedance with hydrodynamic focusing. HGB was measured using sodium lauryl sulfate (SLS) spectrophotometry, and the remaining parameters, such as hematocrit (HCT), mean corpuscular hemoglobin (MCH), and MCH concentration (MCHC), were computed from the parameters listed above. WBC and differential counts were determined using fluorescence flow cytometry.

### 2.3 Difference Between Quality Control Mode and Sample Mode

Three concentrations of Bioyuan and XC control materials were analyzed daily in QC and sample modes on both automated hematology analyzers after international QC had been verified. Each measurement was performed twice daily for 15 consecutive days. The following parameters were compared for each concentration of both QC materials across the two measurement modes: WBC, RBC, HGB, HCT, MCV, MCH, MCHC, and PLT. The objective was to determine whether any observed differences in the results between the QC and sample modes were statistically significant.

### 2.4 Difference Between Manual Mode and Autoloader Mode

This part of the experiment was conducted using a Mindray BC‐6800 Plus in QC mode. The hematology analyzer was set in QC mode, and three concentrations of Bioyuan and XC HQCM were analyzed in manual mode (open vial) and autoloader mode (closed vial). Measurements were conducted three times a day for 15 consecutive days, and the WBC, RBC, HGB, HCT, MCV, MCH, MCHC, and PLT counts were compared for each concentration of HQCM in the two measurement modes. The objective was to determine whether any observed differences in the results between the manual and autoloader modes were statistically significant.

### 2.5 Evaluation of Commutability

Twenty‐six blood samples were collected from both healthy individuals and patients of the Yangtze River Shipping General Hospital after processing on 7 November 2025 and were properly transported to the WHCCL once processing was complete. All parameters (WBC, RBC, HGB, and PLT) were assessed in the study, and the measured values fell within the concentration ranges covering those of the HQCM from both brands.

Six QC samples and 26 clinical samples were randomly selected from the testing sequence. Parallel testing was performed on both the BC‐6800 Plus and Sysmex XN‐1000 analyzers. This assay was repeated in triplicate. In each run, the measurement order was randomized and differed from that of the previous round.

Scatter plots were constructed using the results of clinical samples detected from the Sysmex XN‐1000 on the *x*‐axis and those from the BC‐6800 Plus on the *y*‐axis. Deming's regression was used to establish a mathematical relationship between the two measurement procedures. According to the Guideline for evaluation of commutability of reference materials WS/T 356‐2024 [16], commutability assessment was performed using a 95% prediction interval (PI) derived from Deming regression analysis. A QC material was considered commutable with an authentic specimen between the two systems if its mean value, obtained from the BC‐6800 Plus, fell within the PI, indicating the absence of a matrix‐specific effect. Conversely, if the mean value was outside the interval, the material was deemed noncommutable, suggesting the presence of a matrix effect.

### 2.6 Statistical Analysis

Statistical analysis was performed using MedCalc 2022 software (MedCalc Software Ltd., Ostend, Belgium). Data from the QC mode, sample mode, manual mode, and autoloader mode were compared using *t*‐tests, with a *p* value < 0.05 considered statistically significant. The commutability data for the two brands of control materials were analyzed using Microsoft Excel 2016 (Microsoft Co., Redmond, WA, USA).

## 3 Results

### 3.1 Comparison of Detection Results Obtained From the QC Mode and the Sample Mode of Hematology Analyzers

The detection results for two commercial QC materials (Bioyuan and XC) in QC and sample modes were compared using Mindray BC‐6800 Plus and Sysmex XN‐1000 hematology analyzers. On the Mindray BC‐6800 Plus, the measured values for parameters such as WBC, RBC, HGB, and HCT under both modes were recorded (e.g., for low‐concentration Bioyuan: QC mode WBC 3.50 ± 0.11 vs. sample mode 3.40 ± 0.11 × 10^9^/L) (Table 1). Statistical analysis indicated that, for both QC materials at low, medium, and high concentrations, seven parameters (RBC, HGB, HCT, MCV, MCH, MCHC, and PLT) showed no statistically significant differences (*p* > 0.05) between the two modes. However, for WBC, a significant difference (*p* < 0.05) was observed for the Bioyuan material at all three concentrations, whereas for the XC material, it was only significant at low concentrations.

**TABLE 1:** Statistical comparison of the results for two types of control materials measured in the QC mode versus the sample mode on the BC‐6800 hematology analyzer (*n* = 30).

Instruments	QC materials	Concentrations	Parameters	QC mode		Sample mode		*t*	*p*
				Mean	SD	Mean	SD		
Mindray BC‐6800 Plus	Bioyuan	Low	WBC (10^9^/L)^a^	3.50	0.11	3.40	0.11	4.58	< 0.001
			RBC (10^12^/L)	2.60	0.04	2.59	0.05	3.85	0.05
			HGB (g/L)	73.40	0.72	73.27	0.83	0.11	0.75
			HCT (%)	23.13	0.32	22.98	0.45	3.39	0.07
			MCV (fL)	88.90	0.34	88.88	0.35	0.05	0.82
			MCH (pg)	28.21	0.22	28.29	0.36	1.77	0.19
			MCHC (g/L)	317.07	0.42	318.93	0.75	0.73	0.40
			PLT (10^9^/L)	87.80	2.58	86.47	4.04	2.94	0.09
		Middle	WBC (10^9^/L)^a^	7.86	0.15	7.58	0.15	75.19	< 0.001
			RBC (10^12^/L)	4.66	0.06	4.65	0.05	0.19	0.67
			HGB (g/L)	136.74	1.00	136.42	0.94	1.31	0.19
			HCT (%)	44.66	0.52	44.55	0.56	0.46	0.74
			MCV (fL)	95.91	0.46	95.81	0.42	0.88	0.38
			MCH (pg)	29.83	0.38	29.77	0.35	0.60	0.55
			MCHC (g/L)	310.87	3.68	310.60	4.01	0.27	0.79
			PLT (10^9^/L)	208.43	5.81	207.63	5.87	0.53	0.60
		High	WBC (10^9^/L)^a^	17.08	0.27	16.77	0.30	27.54	< 0.001
			RBC (10^12^/L)	5.59	0.08	5.60	0.07	0.89	0.35
			HGB (g/L)	166.33	1.18	166.27	0.87	0.25	0.80
			HCT (%)	55.22	0.83	55.41	0.79	0.91	0.37
			MCV (fL)	98.85	0.64	98.89	0.63	0.22	0.82
			MCH (pg)	29.76	0.25	29.67	0.32	1.22	0.23
			MCHC (g/L)	301.10	2.86	300.07	3.47	1.26	0.21
			PLT (10^9^/L)	448.03	12.00	449.13	13.72	0.33	0.74
	XC	Low	WBC (10^9^/L)^a^	3.38	0.06	3.27	0.07	6.50	< 0.001
			RBC (10^12^/L)	2.75	0.02	2.76	0.03	0.14	0.89
			HGB (g/L)	46.93	0.46	47.20	0.41	1.67	0.11
			HCT (%)	18.21	0.16	18.19	0.20	0.42	0.68
			MCV (fL)	66.09	0.15	66.04	0.27	0.89	0.38
			MCH (pg)	17.07	0.22	17.15	0.18	1.52	0.14
			MCHC (g/L)	257.67	3.74	259.20	2.92	1.77	0.87
			PLT (10^9^/L)	101.53	7.63	104.88	7.25	1.77	0.08
		Middle	WBC (10^9^/L)	8.82	0.09	8.96	0.17	1.09	0.28
			RBC (10^12^/L)	5.28	0.04	5.28	0.05	0.15	0.88
			HGB (g/L)	95.29	0.46	95.13	0.34	1.69	0.10
			HCT (%)	33.99	0.27	33.94	0.33	0.68	0.50
			MCV (fL)	64.27	0.40	64.25	0.33	0.23	0.82
			MCH (pg)	18.01	0.12	18.23	0.13	0.44	0.66
			MCHC (g/L)	280.29	2.89	280.31	2.29	0.03	0.98
			PLT (10^9^/L)	209.24	9.10	206.44	7.90	1.33	0.19
		High	WBC (10^9^/L)	12.56	0.12	12.59	0.17	0.86	0.39
			RBC (10^12^/L)	5.83	0.05	5.81	0.05	1.50	0.14
			HGB (g/L)	109.13	0.71	109.12	0.33	0.05	0.96
			HCT (%)	39.77	0.35	39.64	0.35	1.54	0.13
			MCV (fL)	68.24	0.19	68.25	0.24	0.18	0.86
			MCH (pg)	18.71	0.15	18.79	0.18	1.99	0.05
			MCHC (g/L)	274.38	2.21	275.29	2.43	1.60	0.11
			PLT (10^9^/L)	252.88	8.43	251.82	9.32	0.48	0.63

Abbreviations: *p*, bilateral probability; SD, standard deviation.

^a^ The difference in the results of this parameter between the two measurement modes is statistically significant.

On the Sysmex XN‐1000, the measured results for both materials under the two modes were obtained (e.g., for high‐concentration Bioyuan: QC mode WBC 16.58 ± 0.47 vs. sample mode 16.12 ± 0.28 × 10^9^/L) (Table 2). The analysis revealed a consistent pattern of significant differences (*p* < 0.05) between the QC and sample modes for WBC count, HCT and MCV across all concentrations of both materials. In contrast, no significant differences (*p* > 0.05) were observed in RBC, HGB, MCH, or PLT at any concentration. For the MCHC, significant differences were observed for the XC material at all concentrations and for the Bioyuan material at medium and high concentrations, but not at low concentrations.

**TABLE 2:** Statistical comparison of the results for two types of control materials measured in the quality control mode versus the sample mode on the XN‐1000 hematology analyzer (*n* = 30).

Instruments	QC materials	Concentrations	Parameters	QC mode		Sample mode		*t*	*p*
				Mean	SD	Mean	SD		
Sysmex XN‐1000	Bioyuan	Low	WBC (10^9^/L)^a^	3.52	0.08	3.49	0.05	2.05	0.04
			RBC (10^12^/L)	2.54	0.02	2.55	0.03	1.82	0.07
			HGB (g/L)	68.15	0.67	68.30	0.47	1.07	0.29
			HCT (%)^a^	20.61	0.27	20.92	0.33	3.95	< 0.001
			MCV (fL)^a^	81.76	0.26	81.33	0.56	4.22	< 0.001
			MCH (pg)	26.84	0.26	26.72	0.25	1.81	0.08
			MCHC (g/L)	326.90	2.95	327.30	4.40	0.41	0.68
			PLT (10^9^/L)	76.97	3.90	78.10	2.71	1.31	0.20
		Middle	WBC (10^9^/L)^a^	7.53	0.13	7.74	0.18	5.32	< 0.001
			RBC (10^12^/L)	4.63	0.05	4.62	0.06	0.52	0.60
			HGB (g/L)	136.74	1.00	136.42	0.94	1.31	0.19
			HCT (%)^a^	44.87	0.56	44.20	0.72	4.17	< 0.001
			MCV (fL)^a^	96.87	0.37	95.57	0.84	7.95	< 0.001
			MCH (pg)	29.52	0.28	29.51	0.35	0.13	0.90
			MCHC (g/L)^a^	304.84	3.29	308.76	4.40	4.01	< 0.001
			PLT (10^9^/L)	203.68	6.01	201.06	8.38	1.43	0.16
		High	WBC (10^9^/L)^a^	16.58	0.47	16.12	0.28	4.76	< 0.001
			RBC (10^12^/L)	5.55	0.05	5.57	0.05	1.27	0.21
			HGB (g/L)	167.05	1.34	167.16	0.83	0.47	0.64
			HCT (%)^a^	55.96	0.59	55.33	0.72	3.60	< 0.001
			MCV (fL)^a^	100.82	0.38	99.39	0.74	9.08	< 0.001
			MCH (pg)	29.74	0.23	29.73	0.25	0.13	0.89
			MCHC (g/L)^a^	296.07	3.13	298.03	3.15	2.40	0.02
			PLT (10^9^/L)	443.52	11.31	437.33	15.57	1.72	0.09
	XC	Low	WBC (10^9^/L)^a^	3.11	0.09	3.17	0.08	2.88	0.01
			RBC (10^12^/L)	2.65	0.03	2.63	0.06	1.82	0.07
			HGB (g/L)	45.93	0.72	46.00	0.80	0.36	0.72
			HCT (%)^a^	15.95	0.19	15.65	0.33	4.19	< 0.001
			MCV (fL)^a^	60.09	0.28	59.20	0.44	9.36	< 0.001
			MCH (pg)	17.40	0.40	17.46	0.33	0.67	0.50
			MCHC (g/L)^a^	287.93	4.51	294.81	4.78	5.71	< 0.001
			PLT (10^9^/L)	94.70	6.84	95.40	9.31	0.33	0.74
		Middle	WBC (10^9^/L)^a^	8.65	0.08	8.70	0.10	2.21	0.03
			RBC (10^12^/L)	5.19	0.05	5.20	0.06	0.54	0.59
			HGB (g/L)	93.80	0.76	93.80	0.85	< 0.001	1.00
			HCT (%)^a^	29.25	0.37	29.00	0.42	0.74	0.02
			MCV (fL)^a^	56.47	0.25	55.76	0.46	7.40	< 0.001
			MCH (pg)	18.06	0.19	18.05	0.25	0.24	0.81
			MCHC (g/L)^a^	319.87	3.52	323.47	4.58	3.42	< 0.001
			PLT (10^9^/L)	174.47	10.29	174.93	11.93	0.16	0.87
		High	WBC (10^9^/L)^a^	12.53	0.14	12.78	0.14	6.77	< 0.001
			RBC (10^12^/L)	5.73	0.05	5.73	0.05	0.92	0.36
			HGB (g/L)	109.87	0.51	109.87	0.63	< 0.001	1.00
			HCT (%)^a^	37.33	0.34	36.69	0.40	6.59	< 0.001
			MCV (fL)^a^	65.06	0.39	64.10	0.27	11.03	< 0.001
			MCH (pg)	19.15	0.18	19.20	0.18	1.00	0.32
			MCHC (g/L)^a^	294.27	2.72	299.53	3.82	6.15	< 0.001
			PLT (10^9^/L)	193.18	9.58	191.72	9.57	0.80	0.43

Abbreviations: *p*, bilateral probability; SD, standard deviation.

^a^ The difference in the results of this parameter between the two measurement modes is statistically significant.

### 3.2 A Comparison of Detection Results Obtained From Manual Mode and Autoloader Mode of Mindray BC‐6800 Plus Hematology Analyzer

The results of the two QC materials obtained under manual (open vial) and autoloader (closed vial) loading modes were compared, whereas Mindray BC‐6800 Plus was operated in QC mode. The measured values of the eight parameters (WBC, RBC, HGB, HCT, MCV, MCH, MCHC, and PLT) in both loading modes were documented. For instance, for low‐concentration Bioyuan material, the manual mode results were WBC 3.53 ± 0.10 × 10^9^/L, RBC 2.57 ± 0.03 × 10^12^/L, HGB 74.00 ± 1.00 g/L, and HCT 23.40% ± 0.20%, whereas autoloader mode results were WBC 3.49 ± 0.11 × 10^9^/L, RBC 2.63 ± 0.02 × 10^12^/L, HGB 73.00 ± 1.00 × g/L, and HCT 22.90% ± 0.30% (Table 3). Statistical comparisons showed that for most parameters, such as WBC, RBC, HGB, HCT, MCH, and MCHC, statistically significant differences (*p* < 0.05) existed between the manual and autoloader modes. The results showed that for the Bioyuan material, MCV showed no significant difference at any concentration. For the XC material, PLT showed no difference at any concentration, and MCV showed no difference, specifically at the medium concentration.

**TABLE 3:** Statistical comparison of the results for two types of control materials measured under the manual mode versus the autoloader mode on the BC‐6800 Plus hematology analyzer (*n* = 45).

Instruments	QC materials	Concentrations	Parameters	Manual mode		Autoloader mode		*t*	*p*
				Mean	SD	Mean	SD		
Mindray BC‐6800 Plus	Bioyuan	Low	WBC (10^9^/L)^a^	3.53	0.10	3.49	0.11	1.99	0.04
			RBC (10^12^/L)^a^	2.57	0.03	2.63	0.02	10.20	< 0.001
			HGB (g/L)^a^	74.00	1.00	73.00	1.00	8.08	< 0.001
			HCT (%)^a^	23.40	0.20	22.90	0.30	10.07	< 0.001
			MCV (fL)	88.90	0.30	88.80	0.40	1.35	0.18
			MCH (pg)^a^	28.10	0.30	28.30	0.30	3.35	< 0.001
			MCHC (g/L)^a^	316.00	3.00	318.00	3.00	4.25	< 0.001
			PLT (10^9^/L)^a^	87.00	4.00	89.00	3.00	2.81	0.01
		Medium	WBC (10^9^/L)^a^	7.80	0.12	7.92	0.16	4.00	< 0.001
			RBC (10^12^/L)^a^	4.61	0.04	4.70	0.03	13.01	< 0.001
			HGB (g/L)^a^	139.00	1.00	139.00	1.00	2.07	0.04
			HCT (%)^a^	45.10	0.40	44.20	0.40	11.02	< 0.001
			MCV (fL)	95.90	0.50	95.90	0.50	0.02	0.98
			MCH (pg)^a^	29.50	0.20	30.20	0.20	14.85	< 0.001
			MCHC (g/L)^a^	307.00	2.00	314.00	2.00	15.93	< 0.001
			PLT (10^9^/L)^a^	204.00	6.00	213.00	6.00	7.00	< 0.001
		High	WBC (10^9^/L)^a^	16.96	0.28	17.21	0.20	5.00	< 0.001
			RBC (10^12^/L)^a^	5.65	0.04	5.52	0.05	13.36	< 0.001
			HGB (g/L)^a^	167.00	1.00	165.00	1.00	7.05	< 0.001
			HCT (%)^a^	55.90	0.60	54.50	0.60	10.79	< 0.001
			MCV (fL)	99.00	0.60	98.70	0.60	1.55	0.13
			MCH (pg)^a^	30.00	0.20	29.60	0.20	9.32	< 0.001
			MCHC (g/L)^a^	303.00	2.00	299.00	2.00	8.79	< 0.001
			PLT (10^9^/L)^a^	437.00	9.00	458.00	10.00	10.31	< 0.001
	XC	Low	WBC (10^9^/L)^a^	3.23	0.09	3.10	0.10	5.16	< 0.001
			RBC (10^12^/L)^a^	2.62	0.03	2.56	0.02	9.27	< 0.001
			HGB (g/L)^a^	47.06	0.25	46.69	0.47	3.99	< 0.001
			HCT (%)^a^	16.52	0.16	16.22	0.15	7.76	< 0.001
			MCV (fL)^a^	63.03	0.28	63.26	0.28	3.24	< 0.001
			MCH (pg)^a^	17.94	0.15	18.20	0.16	6.67	< 0.001
			MCHC (g/L)^a^	284.91	2.60	287.94	4.06	3.55	< 0.001
			PLT (10^9^/L)	157.84	7.07	158.72	7.91	0.47	0.64
		Medium	WBC (10^9^/L)^a^	6.82	0.12	6.57	0.14	7.63	< 0.001
			RBC (10^12^/L)^a^	4.35	0.03	4.25	0.03	12.86	< 0.001
			HGB (g/L)^a^	83.48	0.51	83.09	0.30	3.77	< 0.001
		
			HCT (%)^a^	31.24	0.24	30.53	0.20	12.56	< 0.001
			MCV (fL)	71.78	0.25	71.84	0.29	0.89	0.38
			MCH (pg)^a^	19.19	0.10	19.60	0.14	13.48	< 0.001
			MCHC (g/L)^a^	267.34	1.88	272.00	1.95	9.73	< 0.001
		PLT (10^9^/L)	191.00	9.16	191.66	8.78	0.30	0.77
		High	WBC (10^9^/L)^a^	13.42	0.22	13.14	0.20	5.30	< 0.001
			RBC (10^12^/L)^a^	5.90	0.04	5.79	0.04	10.44	< 0.001
			HGB (g/L)^a^	109.06	0.25	111.09	0.39	24.50	< 0.001
			HCT (%)^a^	36.37	0.36	35.59	0.40	8.09	< 0.001
			MCV (fL)^a^	61.65	0.38	61.44	0.40	2.14	0.04
			MCH (pg)^a^	18.52	0.17	19.18	0.16	15.91	0.01
			MCHC (g/L)^a^	300.16	3.49	312.03	3.84	12.82	< 0.001
PLT (10^9^/L)	324.23		12.35	328.41	11.79	1.37	0.17

Abbreviations: *p*, bilateral probability; SD, standard deviation.

^a^ The difference in the results for this parameter between the two modes was statistically significant.

### 3.3 Evaluation of Commutability

The commutability of commercial QC materials with clinical patient samples was evaluated for four directly measured parameters (WBC, RBC, HGB, and PLT) using the 95% PI method based on Deming regression between the Sysmex XN‐1000 (reference) and Mindray BC‐6800 Plus (test) systems. The measured values of the QC materials in the test system were plotted against the regression lines of the clinical samples. For example, the Bioyuan QC material results on the test system were WBC 3.50, 7.76, 16.77 × 10^9^/L; RBC 2.62, 4.67, 5.63 × 10^12^/L; HGB 72.67, 135.48, 166.86 × g/L; and PLT 75.67, 213.67, 462.67 × 10^9^/L for low, medium, and high concentrations, respectively (Table 4).

**TABLE 4:** Evaluation of the commutability between two commercial quality control materials and clinical samples.

QC materials	Parameters	Sysmex XN‐1000	Mindray BC‐6800 plus	Predicted value	Lower limit of 95% PI	Upper limit of 95% PI	Commutable
Bioyuan L	WBC (10^9^/L)	3.45	3.50	3.43	3.14	3.73	Y
Bioyuan M		7.72	7.76	7.79	7.49	8.08	Y
Bioyuan H		15.77	16.77	16.00	15.70	16.31	N
XC L		3.14	3.21	3.11	2.82	3.40	Y
XC M		8.55	8.73	8.64	8.34	8.94	Y
XC H		12.65	12.87	12.82	12.52	13.13	Y
Bioyuan L	RBC (10^12^/L)	2.57	2.62	2.67	2.61	2.73	Y
Bioyuan M		4.62	4.67	4.64	4.58	4.70	Y
Bioyuan H		5.51	5.63	5.49	5.43	5.55	N
XC L		2.59	2.74	2.68	2.63	2.74	Y
XC M		5.23	5.31	5.22	5.16	5.28	N
XC H		5.69	5.77	5.67	5.61	5.72	N
Bioyuan L	PLT (10^9^/L)	75.33	75.67	74.42	31.74	117.00	Y
Bioyuan M		223.33	213.67	224.57	181.91	267.24	Y
Bioyuan H		460.00	462.67	464.67	422.03	507.51	Y
XC L		78.33	84.33	77.47	34.79	120.04	Y
XC M		181.67	185.33	182.30	139.64	224.95	Y
XC H		199.67	247.33	200.57	157.90	243.22	N
Bioyuan L	HGB (g/L)	69.33	72.67	70.33	68.98	71.64	N
Bioyuan M		133.67	135.48	134.30	132.98	135.61	Y
Bioyuan H		166.60	166.86	167.04	165.73	168.35	Y
XC L		46.00	47.00	47.14	45.77	48.45	Y
XC M		93.33	95.00	94.19	92.85	95.51	Y
XC H		111.67	109.00	112.43	111.10	113.74	N

*Note:* L, M, and H represent low, medium, and high concentrations of QC materials, respectively. Y indicates that the parameters of the commercial material are commutable to the samples. N indicates no, denoting noncommutability.

Abbreviation: PI, prediction interval.

The assessment revealed that commutability was both parameter‐ and concentration‐dependent. For the Bioyuan material, the PLT results at all three concentrations were within 95% PI, indicating commutability. The WBC and RBC results were commutable at low and medium concentrations but exceeded the PI at high concentrations. HGB was commutable at medium and high concentrations but exceeded the PI at low concentrations. For XC, the WBC counts at all concentrations were within the PI (Figure 1A). RBC results were detectable only at low concentrations (Figure 1B). PLT and HGB were commutable at low and medium concentrations; at high concentrations, PLT exceeded the upper PI limit, and HGB fell below the lower limit (Figure 1C,D).

**FIGURE 1:**
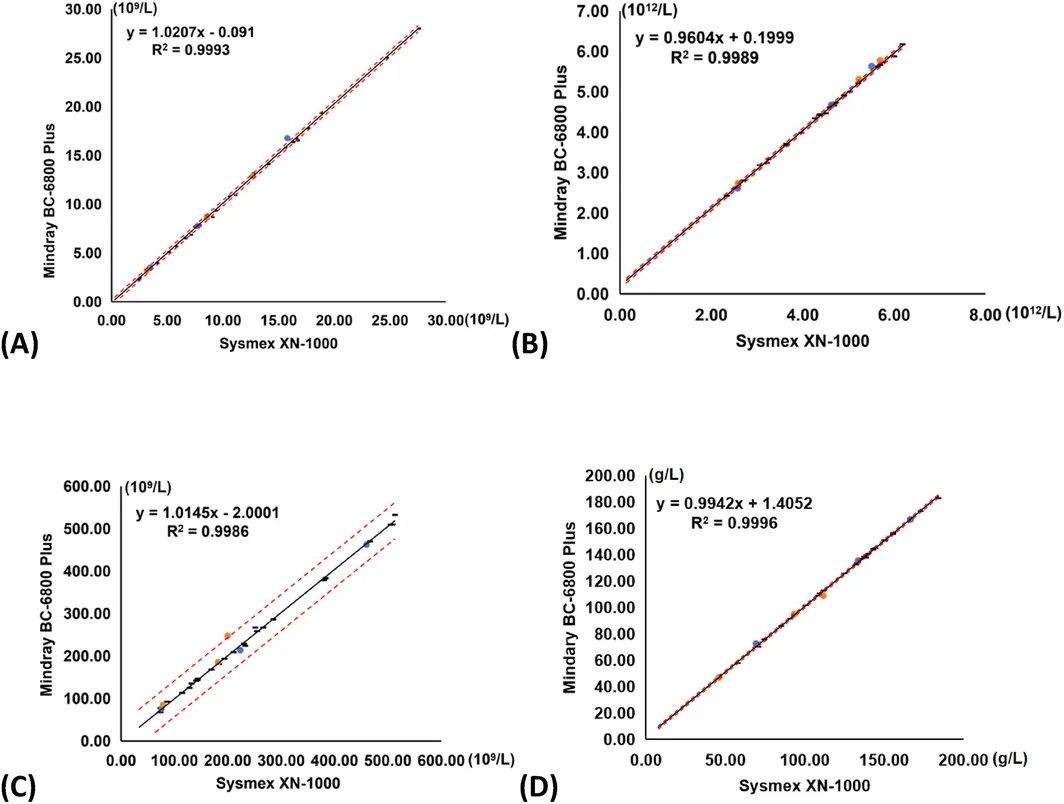
The commutability assessment for WBC, RBC, PLT, and HGB. (A–D) Deming regression plot for the parameters WBC, RBC, PLT, and HGB, respectively. Blue circles represent the results of Bioyuan QC material, and red circles denote these of XC QC material. The thick short black straight lines indicate clinical samples, whereas the long solid lines represent the Deming regression lines. The two red dashed lines correspond to the upper and lower limits of the 95% prediction interval, respectively.

## 4 Discussion

As a routine test, CBC plays a vital role in clinical diagnosis and treatment. Therefore, ensuring the accuracy of blood cell analysis results is essential for daily work in a clinical laboratory [4, 17]. Within a comprehensive QC process, QC materials are indispensable key substances that must be commutable [13, 18, 19]. However, a critical challenge lies in the potential for matrix effects in commercial QC materials, which may not behave identically to fresh patient samples across different analytical systems, thus risking erroneous clinical decision‐making. This underscores the necessity for the rigorous evaluation of QC material performance to safeguard the integrity of hematological testing.

This study systematically investigated the analytical performance and commutability of commercial HQCM using two standard automated analyzers. We focused on evaluating the differences induced by instrument measurement modes (QC vs. sample), sampling modes (manual vs. autoloader), and commutability of materials between systems, as noncommutable controls can lead to significant measurement biases and potential clinical errors.

A key question in this process is whether QC materials should be analyzed using the QC or sample mode. According to the guideline requirements [20], QC materials should be tested using the same detection protocol as clinical samples, implying that they should be analyzed in the sample mode. However, for commercial CBC QC materials not produced by the instrument manufacturer, various chemical substances are often added during production to extend the shelf life and maintain stability [4]. These additives increase the differences between the QC material and specimens, leading to abnormal results when the QC materials are tested in the sample mode. Consequently, QC manufacturers often recommend the use of a dedicated QC mode to test their products. This study systematically investigated whether the results differed between the QC and sample modes for commercial QC materials from two different manufacturers. The results revealed that the difference between the two modes was primarily observed in WBC. The Bioyuan QC materials, derived from human blood sources, showed significant differences from the whole‐blood samples due to their additives. The XC QC materials, sourced from porcine blood, are inherently different from human blood samples. When analyzed in sample mode using blood cell analyzers, the instrument processes these as if they were clinical samples. By contrast, the QC mode uses proprietary internal algorithms designed to account for the specific properties of QC materials, thereby optimizing the measured values for a reliable quality assessment. Thus, a systematic discrepancy in WBC count was observed between the two modes on both Mindray BC‐6800 Plus and Sysmex XN‐1000 hematology analyzers.

For RBC, PLT, HGB and MCH, the results showed no differences between the two modes across all three concentration levels of both QC materials in either analyzers. This consistency can be attributed to the analytical principles involved; both analyzers determine RBC and PLT counts on the basis of the Coulter principle, which involves counting cell particles in the same manner, regardless of the mode. Similarly, the determination of HGB by both instruments relied on the same colorimetric method, and data processing was consistent between modes, leading to the absence of differences in HGB and MCH. Differences in MCV, MCHC, and HCT were observed between the two modes on the Sysmex XN‐1000, but not on the Mindray BC‐6800 Plus. This discrepancy is likely due to the differences in the internal algorithms and data handling specific to each analyzer. Concurrently, we compared the coefficient of variation (CV) values (Tables S3 and S4) between the control and sample modes for each parameter. In both detection systems, the CV values for most parameters in the control mode were lower than those in the sample mode, indicating that third‐party control materials should be tested in the control mode.

WBC had significant inter‐mode differences on both analyzers, with mean deviations of 0.10–0.46 × 10^9^/L. Within the ±10% allowable error, such deviations are safe for routine cases, but cumulative errors from frequent mode switching may disturb internal QC. RBC, HGB, and PLT showed no significant differences, with deviations well below clinical limits (RBC ±6%, HGB ±5%, PLT ±15%), ensuring reliable diagnosis of anemia and platelet disorders. On XN‐1000, HCT (0.29%–0.67%) and MCV (0.4–1.43 fL) differed obviously between modes. Deviations are acceptable for routine anemia classification but may affect diagnosis of hereditary erythrocyte and iron metabolism diseases. BC‐6800 Plus showed stable HCT and MCV results across modes. MCH had no clinical impact. MCHC differed significantly on XN‐1000 (3.92–5.26 g/L), which does not cause misdiagnosis but interferes with long‐term QC trend analysis. Most parameters showed larger deviations between the two sampling modes. Deviations of WBC, RBC, HGB and HCT nearly reached the allowable error limit. Random mode switching causes abnormal QC fluctuations, false out‐of‐control alarms and inconsistent results for clinical samples. MCV was stable across sampling modes and unaffected for anemia classification. Fluctuations of MCH and MCHC are secondary to RBC and HGB changes and impair comprehensive erythrocyte evaluation. PLT of Bioyuan materials varied significantly between modes, bringing risks for patients with coagulation disorders and chemotherapy patients.

Although hematology analyzers can process both urgent and routine samples, their handling differs; urgent samples, after being mixed and opened, are typically analyzed in manual mode, whereas routine samples are usually processed automatically via a sample rack without opening the tubes. This approach improves workflow efficiency but requires prior verification by laboratory technicians to ensure the consistency of clinical sample results between these two loading modes [4, 5]. Comparisons of hematologic modes of use are a critical step in the performance verification of analyzers [21, 22]. Manufacturers recommend the use of a dedicated QC mode for testing. However, it remains unclear whether the detection results for QC materials differ between manual and autoloader modes within this QC mode. This study revealed that for the Mindray BC‐6800 Plus analyzer operating in QC mode, most parameters (such as WBC, RBC, HGB, HCT, MCH and MCHC) showed differences between the two loading modes for the QC materials tested. The exceptions were the MCV across all three levels of the Bio yuan QC material, MCV at the medium level of the XC material and PLT across all three levels of the XC material, which showed no significant differences. Furthermore, the CV values for the parameters showing differences between the two modes were compared (Table S5), and no significant differences in CV were found. This suggests that for daily QC practices, either manual or automatic loading in QC mode is acceptable, provided that a single loading method is consistently used for a given QC material lot.

As mentioned above, commercial QC materials for international QC in hematological analyses should exhibit properties comparable to or similar to those of clinical samples, a characteristic known as commutability. The ISO 17043:2023 standard also emphasizes the commutability of the proficiency testing materials used in EQA schemes [23]. However, in developing countries such as China, acquiring highly commutable commercial QC materials is challenging because of their high cost and short shelf life, limiting their widespread use in numerous laboratories and large‐scale EQA programs. Consequently, several medical laboratories and EQA providers opted for more affordable alternatives. However, research specifically evaluating the commutability of hematological QC materials available in China is lacking. This study was designed to fill this gap. According to WS/T 356‐2024, we assessed the commutability of two brands of commercial QC materials at high, medium, and low concentrations. The findings indicated that only some parameters of each QC material demonstrated commutability with clinical samples, whereas others exhibited matrix effects. Studies [24, 25, 26] have found a key limitation of the 95% PI approach of commutability evaluation: it is more stringent for a more precise method but less stringent for a less precise method, thereby allowing a reference material to be classified as commutable for a method with poor performance characteristics, but noncommutable for a method with good performance characteristics. As shown in Table S6, the Mindray BC‐6800 Plus and Sysmex XN‐1000 analytical systems employed in our laboratory demonstrated good precision performance, complying with the requirements of the analytical performance standards. Notably, the precision of the RBC was approximately 1% for both systems. We hypothesized that this high level of precision might be the reason for the observed noncommutability of the two QC materials for RBC measurement. Another method for evaluating the commutability of reference materials was recommended by the International Federation of Clinical Chemistry and Laboratory Medicine (IFCC) [27], and one advantage of this method is its ability to quantify the closeness between reference materials and clinical samples, as well as the uncertainty of that closeness; therefore, it is considered clinically more valuable than the guidance of CLSI25. Non‐commutability was mainly observed at extreme concentrations. Low‐concentration non‐commutable materials (for severe anemia) fail to reflect real instrument performance in cross‐platform QC. High‐concentration materials (for leukemia, polycythemia, and severe thrombocytosis) cannot simulate fresh clinical samples, leading to biased EQA and inter‐laboratory results and obstructing test standardization. Overall, detected differences are mostly acceptable for routine diagnosis, but negatively affect critical patient monitoring, QC, cross‐instrument alignment, and EQA. Standardized operating protocols and commutable QC materials are required to ensure testing accuracy and quality management.

Although this study provides valuable insights, several limitations warrant consideration. The commutability assessment relied on a relatively small set of clinical samples (*n* = 26), which may have affected the robustness of the Deming regression and the derived PIs. Furthermore, the investigation was conducted under controlled research conditions. Thus, the generalizability of the findings to high‐throughput, real‐world clinical laboratories with diverse operators and sample matrices requires validation. The evaluation was also restricted to specific models from the two analyzer brands, limiting extrapolation to other platforms. Finally, this study did not mechanistically explore the underlying causes of the observed mode‐dependent differences or matrix effects.

In summary, this systematic evaluation underscores the influence of multiple analytical variables on the reliability of hematological QC. The key findings demonstrate that the measurement mode (QC vs. sample) can introduce significant analyzer‐dependent biases, particularly for WBC and calculated parameters. Moreover, the sampling method (manual versus autoloader) was a major source of variability for most parameters. Commercially available QC materials frequently exhibit noncommutability, especially at extreme concentrations, indicating significant matrix effects that challenge their use for direct inter‐instrument comparison. These results emphasize the necessity for laboratories to standardize testing protocols, critically select and assign QC materials, and interpret QC data with awareness of these influencing factors to ensure accurate and harmonized patient results.

## Author Contributions

Bingshuang Ren, Ya Cheng, and Hongping Ba were involved in investigation, formal analysis, and writing‐original draft. Weijia Cheng and Xuwei Zhou were involved in investigation and resources. Hui Zhen was involved in investigation. Jie Gong and Shimin Wu were involved in writing‐review, resources, and supervision. Author agreement: All authors have seen and approved the final version of the manuscript submitted and confirm that this manuscript has not been previously published and is not currently under consideration by any other journal.

## Funding

This study was supported by the funding for Scientific Research Projects from Wuhan Municipal Health Commission (WX23Z42 and WX23B51).

## Ethics Statement

The use of these anonymized residual samples for method comparison was approved by the General Hospital of The Yangtze River Shipping Ethics Committee (Ethics Approval No.: YL20250062) and the Wuhan Center for Clinical Laboratory (WHCCL) Ethics Committee (Ethics Approval No.: KY2025001), and this research was conducted in accordance with the ethical principles of the Declaration of Helsink.

## Consent

A total of 26 clinical whole blood samples were obtained from the General Hospital of The Yangtze River Shipping, all 26 patients have signed the informed consent form.

## Conflicts of Interest

The authors declare no conflicts of interest.

## Supporting information


**Table S1:** Target values of eight parameters of Bioyuan HQCM provided by manufacturer.
**Table S2:** Target values of five parameters of XC HQCM provided by manufacturer.
**Table S3:** Coefficients of variation (CV) under the sample mode and the quality control mode of the Mindray BC‐6800 Plus by analazing HQCM.
**Table S4:** Coefficients of variation (CV) under the sample mode and the quality control mode of the Sysmex XN‐1000 by analazing HQCM.
**Table S5:** Coefficients of variation (CV) under the manual mode and the autoloader mode of the Mindray BC‐6800 Plus by analazing HQCM
**Table S6:** IQC data obtained using the instrument manufacturer‐provided QC materials.

## Data Availability

The data that support the findings of this study are available from the corresponding author upon reasonable request.

## References

[1] J. Ha , M. Ono , H. Zhu , C. Cencer , X. Ge , and J. Y. Wu , “Complete Blood Count Parameters and Bone Health: Clinical and Experimental Evidence,” Endocrinology and Metabolism 40, no. 6 (2025): 811–820. 10.3803/EnM.2025.2695 PMC12765876 41331963

[2] C. H. Wolfsberger , B. N. Seidel , L. Fleck , et al., “Complete Blood Count Reference Intervals for Extremely Preterm Neonates,” European Journal of Pediatrics 184, no. 11 (2025): 699. 10.1007/s00431-025-06544-4 PMC12535521 41108422

[3] E. Hyde , M. Anthony , S. Kennedy , and M. Vatish , “Role of Complete Blood Count in the Diagnosis of Culture‐Proven Neonatal Sepsis: A Systematic Review and Meta‐Analysis,” Archives of Disease in Childhood 110, no. 10 (2025): 811–818. 10.1136/archdischild-2025-328523 PMC12505116 40412822

[4] G. S. Cembrowski and G. Clarke , “Quality Control of Automated Cell Counters,” Clinics in Laboratory Medicine 35, no. 1 (2015): 59–71. 10.1016/j.cll.2014.10.006 25676372

[5] J. Y. Vis and A. Huisman , “Verification and Quality Control of Routine Hematology Analyzers,” International Journal of Laboratory Hematology 38, no. Suppl 1 (2016): 100–109. 10.1111/ijlh.12503 27161194

[6] S. E. Verbrugge and A. Huisman , “Verification and Standardization of Blood Cell Counters for Routine Clinical Laboratory Tests,” Clinics in Laboratory Medicine 35, no. 1 (2015): 183–196. 10.1016/j.cll.2014.10.008 25676379

[7] ISO 15189 , Medical Laboratories‐Requirements for Quality and Competence (International Organization for Standardization (ISO), 2023).

[8] B. De la Salle , “Survey Material Choices in Haematology EQA: A Confounding Factor in Automated Counting Performance Assessment,” Biochemical Medicine 27, no. 1 (2017): 63–72. 10.11613/BM.2017.008 PMC5382858 28392727

[9] R. McCafferty , G. Cembrowski , B. de la Salle , M. Peng , and E. Urrechaga , “ICSH Guidance for Internal Quality Control Policy for Blood Cell Counters,” International Journal of Laboratory Hematology 46, no. 2 (2024): 227–233. 10.1111/ijlh.14212 38189640

[10] N. N. Vo , H. T. Tran , T. T. N. Le , et al., “Enhanced Stability of Simulated Leukocytes for Hematology Internal Quality Control Samples: A Material Improvement,” PLoS One 20, no. 8 (2025): e0330292. 10.1371/journal.pone.0330292 PMC12352632 40811440

[11] N. N. Vo , H. T. Tran , T. H. P. Nguyen , D. D. Vu , T. S. Vo , and T. H. Nguyen , “Determination of the Assigned Values of Blood Cells by an Impedance Method for Hematological Reference Samples Used in Hematology External Quality Assessment (EQA) Programs,” Biomedicine 10, no. 12 (2022): 3169. 10.3390/biomedicines10123169 PMC9775971 36551923

[12] J. Sioufi , T. Badrick , L. Sinclair , and K. Marsden , “Full Blood Count ‐ Internal QC Protocol: A Review by the Royal College of Pathologists of Australasia Quality Assurance Programs (RCPAQAP) Pty Ltd ‐ Haematology,” International Journal of Laboratory Hematology 39, no. 1 (2017): 84–94. 10.1111/ijlh.12582 27997738

[13] M. Vidali , A. Carobene , S. Apassiti Esposito , et al., “Standardization and Harmonization in Hematology: Instrument Alignment, Quality Control Materials, and Commutability Issue,” International Journal of Laboratory Hematology 43, no. 3 (2021): 364–371. 10.1111/ijlh.13379 33174358

[14] M. Velizarova , T. Yacheva , M. Genova , and D. Svinarov , “Evaluation of Automated Hematology Analyzer DYMIND DH76 Compared to SYSMEX XN 1000 System,” Journal of Medical Biochemistry 40, no. 4 (2021): 367–377. 10.5937/jomb0-28836 PMC8451233 34616226

[15] P. Guo , Q. Cai , M. Mao , et al., “Performance Evaluation of the New Platelet Measurement Channel on the BC‐6800 Plus Automated Hematology Analyzer,” International Journal of Laboratory Hematology 44, no. 2 (2022): 281–287. 10.1111/ijlh.13753 34873856

[16] National Health Commission , Guideline for Evaluation of Commutability of Reference Materials: WS/T 356‐2024, (National Health Commission of the People's Republic of China, 2024).

[17] T. P. Loh , C. Y. Lim , S. K. Sethi , R. Z. Tan , and C. Markus , “Advances in Internal Quality Control,” Critical Reviews in Clinical Laboratory Sciences 60, no. 7 (2023): 502–517. 10.1080/10408363.2023.2209174 37194676

[18] G. R. D. Jones , V. Delatour , and T. Badrick , “Metrological Traceability and Clinical Traceability of Laboratory Results—The Role of Commutability in External Quality Assurance,” Clinical Chemistry and Laboratory Medicine 60, no. 5 (2022): 669–674. 10.1515/cclm-2022-0038 35179002

[19] S. Sandberg , P. Fauskanger , J. V. Johansen , et al., “IFCC Working Group on Commutability in Metrological Traceability. Recommendations for Setting a Criterion and Assessing Commutability of Sample Materials Used in External Quality Assessment/Proficiency Testing Schemes,” Clinical Chemistry 69, no. 11 (2023): 1227–1237. 10.1093/clinchem/hvad135 37725906

[20] International Organization for Standardization (ISO)/International Electrotechnical Commission (IEC) , 15189:2023. Application Requirements of Accreditation Criteria for the Quality and Competence of Medical Laboratories (International Organization for Standardization, 2023).

[21] Clinical Laboratory Standards Institute , H26–A2 Validation, Verification, and Quality Assurance of Automated Hematology Analyzers; Approved Standard Second Edition (Clinical Laboratory Standards Institute, 2010).

[22] Alberta Health Services and Covenant Health , Mode to Mode Hematology Correlation Excel Worksheet (Alberta Health Services, 2014).

[23] International Organization for Standardization (ISO)/International Electrotechnical Commission (IEC) , ISO17043:2023. Conformity Assessment—General Requirements for Proficiency Testing (International Organization for Standardization, 2023).

[24] T. Xing , J. Liu , H. Sun , et al., “Commutability Assessment of Reference Materials for Homocysteine,” Clinical Chemistry and Laboratory Medicine 60, no. 10 (2022): 1562–1569. 10.1515/cclm-2022-0388 35977428

[25] Q. Guo , J. Wang , X. Yi , et al., “Commutability of Reference Materials for Alkaline Phosphatase Measurements,” Scandinavian Journal of Clinical and Laboratory Investigation 80, no. 5 (2020): 388–394. 10.1080/00365513.2020.1747111 32271089

[26] Y. Yan , B. Han , H. Zhao , et al., “Commutability of External Quality Assessment Materials for Serum Sodium and Potassium Measurements,” Clinical Chemistry and Laboratory Medicine 57, no. 4 (2019): 465–475. 10.1515/cclm-2018-0385 30157026

[27] W. G. Miller , H. Schimmel , R. Rej , et al., “IFCC Working Group Recommendations for Assessing Commutability Part 1: General Experimental Design,” Clinical Chemistry 64, no. 3 (2018): 447–454. 10.1373/clinchem.2017.277525 PMC5832613 29348163

