# Supplementary material for: Different Analytical Modes on Automatic Hematology Analyzers for Testing Quality Control Materials and Commutability Assessment
Source: Int J Lab Hematol. 2026 Jul 22;48(6):1365–76. doi: 10.1111/ijlh.70179 (PMC13729927; doi:10.1111/ijlh.70179)
Supplement: Supplementary file 1 [file IJLH-48-1365-s001.docx]

**APPENDIX**

**TABLE S1 Target values of eight parameters of Bioyuan HQCM provided by manufacturer**

| Parameters | Instrument Model | Concentrations/Lot | | |
| --- | --- | --- | --- | --- |
|  |  | Low(253401) | Medium(253402) | High(253403) |
| WBC (10^9^/L) | Sysmex XN-1000 | 3.43 | 7.73 | 16.60 |
|  | Mindray BC-6900 | 3.44 | 7.75 | 16.51 |
| RBC (10^12^/L) | Sysmex XN-1000 | 2.55 | 4.68 | 5.62 |
|  | Mindray BC-6900 | 2.65 | 4.71 | 5.67 |
| HGB (g/L) | Sysmex XN-1000 | 73.50 | 138.75 | 168.50 |
|  | Mindray BC-6900 | 76.00 | 143.67 | 170.83 |
| HCT (%) | Sysmex XN-1000 | 21.70 | 42.43 | 52.90 |
|  | Mindray BC-6900 | 24.45 | 46.23 | 56.98 |
| MCV (fL) | Sysmex XN-1000 | 85.10 | 90.78 | 94.18 |
|  | Mindray BC-6900 | 92.47 | 98.23 | 100.62 |
| MCH (pg) | Sysmex XN-1000 | 28.80 | 29.70 | 30.00 |
|  | Mindray BC-6900 | 28.75 | 30.50 | 30.22 |
| MCHC (g/L) | Sysmex XN-1000 | 338.50 | 327.25 | 318.25 |
|  | Mindray BC-6900 | 311.00 | 310.50 | 300.17 |
| PLT (10^9^/L) | Sysmex XN-1000 | 85.75 | 209.00 | 464.50 |
|  | Mindray BC-6900 | 88.67 | 211.00 | 460.67 |

**TABLE S2 Target values of five parameters of XC HQCM provided by manufacturer**

| Parameters | Instrument Model | Concentrations/Lot | | |
| --- | --- | --- | --- | --- |
|  |  | Low(05250283) | Medium(05250271) | High(05250302) |
| WBC (10^9^/L) | Sysmex KX-21 | 3.70 | 9.20 | 13.10 |
|  | Mindray BC-3000Plus | 3.50 | 8.90 | 12.80 |
| RBC (10^12^/L) | Sysmex KX-21 | 2.83 | 5.44 | 6.17 |
|  | Mindray BC-3000Plus | 2.64 | 5.12 | 5.92 |
| HGB (g/L) | Sysmex KX-21 | 48.00 | 94.00 | 109.00 |
|  | Mindray BC-3000Plus | 48.00 | 94.00 | 109.00 |
| HCT (%) | Sysmex KX-21 | 16.10 | 30.60 | 36.20 |
|  | Mindray BC-3000Plus | 18.20 | 34.80 | 38.60 |
| PLT (10^9^/L) | Sysmex KX-21 | 77.00 | 172.00 | 267.00 |
|  | Mindray BC-3000Plus | 85.00 | 191.00 | 293.00 |

**TABLE S3 Coefficients of variation (CV) under the sample mode and the quality control mode of the Mindray BC-6800 Plus by analazing HQCM**

| HQCM | Concentrations | Parameters | QC Mode CV | Sample Mode CV |
| --- | --- | --- | --- | --- |
| Bioyuan | Low | WBC (10^9^/L) | 3.14% | 3.24% |
|  |  | RBC (10^12^/L) | 1.54% | 1.93% |
|  |  | HGB (g/L) | 0.98% | 1.13% |
|  |  | HCT (%) | 1.38% | 1.96% |
|  |  | MCV (fL) | 0.38% | 0.39% |
|  |  | MCH (pg) | 0.78% | 1.27% |
|  |  | MCHC (g/L) | 0.13% | 0.24% |
|  |  | PLT (10^9^/L) | 2.94% | 4.67% |
|  | Medium | WBC (10^9^/L) | 1.91% | 1.98% |
|  |  | RBC (10^12^/L) | 1.29% | 1.08% |
|  |  | HGB (g/L) | 0.73% | 0.69% |
|  |  | HCT (%) | 1.16% | 1.26% |
|  |  | MCV (fL) | 0.48% | 0.44% |
|  |  | MCH (pg) | 1.27% | 1.18% |
|  |  | MCHC (g/L) | 1.18% | 1.29% |
|  |  | PLT (10^9^/L) | 2.79% | 2.83% |
|  | High | WBC (10^9^/L) | 1.58% | 1.79% |
|  |  | RBC (10^12^/L) | 1.43% | 1.25% |
|  |  | HGB (g/L) | 0.71% | 0.52% |
|  |  | HCT (%) | 1.50% | 1.43% |
|  |  | MCV (fL) | 0.65% | 0.64% |
|  |  | MCH (pg) | 0.84% | 1.08% |
|  |  | MCHC (g/L) | 0.95% | 1.16% |
|  |  | PLT (10^9^/L) | 2.68% | 3.05% |
| XC | Low | WBC (10^9^/L) | 1.78% | 2.14% |
|  |  | RBC (10^12^/L) | 0.73% | 1.09% |
|  |  | HGB (g/L) | 0.98% | 0.87% |
|  |  | HCT (%) | 0.88% | 1.10% |
|  |  | MCV (fL) | 0.23% | 0.41% |
|  |  | MCH (pg) | 1.29% | 1.05% |
|  |  | MCHC (g/L) | 1.45% | 1.13% |
|  |  | PLT (10^9^/L) | 7.52% | 6.91% |
|  | Medium | WBC (10^9^/L) | 1.02% | 1.90% |
|  |  | RBC (10^12^/L) | 0.76% | 0.95% |
|  |  | HGB (g/L) | 0.48% | 0.36% |
|  |  | HCT (%) | 0.79% | 0.97% |
|  |  | MCV (fL) | 0.62% | 0.51% |
|  |  | MCH (pg) | 0.67% | 0.71% |
|  |  | MCHC (g/L) | 1.03% | 0.82% |
|  |  | PLT (10^9^/L) | 4.35% | 3.83% |
|  | High | WBC (10^9^/L) | 0.96% | 1.35% |
|  |  | RBC (10^12^/L) | 0.86% | 0.86% |
|  |  | HGB (g/L) | 0.65% | 0.30% |
|  |  | HCT (%) | 0.88% | 0.88% |
|  |  | MCV (fL) | 0.28% | 0.35% |
|  |  | MCH (pg) | 0.80% | 0.96% |
|  |  | MCHC (g/L) | 0.81% | 0.88% |
|  |  | PLT (10^9^/L) | 3.33% | 3.70% |

**TABLE S4 Coefficients of variation (CV) under the sample mode and the quality control mode of the Sysmex XN-1000 by analazing HQCM**

| HQCM | Concentrations | Parameters | QC Mode CV | Sample Mode CV |
| --- | --- | --- | --- | --- |
| Bioyuan | Low | WBC (10^9^/L) | 2.27% | 1.43% |
|  |  | RBC (10^12^/L) | 0.79% | 1.18% |
|  |  | HGB(g/L) | 0.98% | 0.69% |
|  |  | HCT (%) | 1.31% | 1.58% |
|  |  | MCV (fL) | 0.32% | 0.69% |
|  |  | MCH (pg) | 0.97% | 0.94% |
|  |  | MCHC (g/L) | 0.90% | 1.34% |
|  |  | PLT (10^9^/L) | 5.07% | 3.47% |
|  | Medium | WBC (10^9^/L) | 1.73% | 2.33% |
|  |  | RBC (10^12^/L) | 1.08% | 1.30% |
|  |  | HGB (g/L) | 0.73% | 0.69% |
|  |  | HCT (%) | 1.25% | 1.63% |
|  |  | MCV (fL) | 0.38% | 0.88% |
|  |  | MCH (pg) | 0.95% | 1.19% |
|  |  | MCHC (g/L) | 1.08% | 1.43% |
|  |  | PLT (10^9^/L) | 2.95% | 4.17% |
|  | High | WBC (10^9^/L) | 2.83% | 1.74% |
|  |  | RBC (10^12^/L) | 0.90% | 0.90% |
|  |  | HGB (g/L) | 0.80% | 0.50% |
|  |  | HCT (%) | 1.05% | 1.30% |
|  |  | MCV (fL) | 0.38% | 0.74% |
|  |  | MCH (pg) | 0.77% | 0.84% |
|  |  | MCHC (g/L) | 1.06% | 1.06% |
|  |  | PLT (10^9^/L) | 2.55% | 3.56% |
| XC | Low | WBC (10^9^/L) | 2.89% | 2.52% |
|  |  | RBC (10^12^/L) | 1.13% | 2.28% |
|  |  | HGB (g/L) | 1.57% | 1.74% |
|  |  | HCT (%) | 1.19% | 2.11% |
|  |  | MCV (fL) | 0.47% | 0.74% |
|  |  | MCH (pg) | 2.30% | 1.89% |
|  |  | MCHC (g/L) | 1.57% | 1.62% |
|  |  | PLT (10^9^/L) | 7.22% | 9.76% |
|  | Medium | WBC (10^9^/L) | 0.92% | 1.15% |
|  |  | RBC (10^12^/L) | 0.96% | 1.15% |
|  |  | HGB (g/L) | 0.81% | 0.91% |
|  |  | HCT (%) | 1.26% | 1.45% |
|  |  | MCV (fL) | 0.44% | 0.82% |
|  |  | MCH (pg) | 1.05% | 1.39% |
|  |  | MCHC (g/L) | 1.10% | 1.42% |
|  |  | PLT (10^9^/L) | 5.90% | 6.82% |
|  | High | WBC (10^9^/L) | 1.12% | 1.10% |
|  |  | RBC (10^12^/L) | 0.87% | 0.87% |
|  |  | HGB (g/L) | 0.46% | 0.57% |
|  |  | HCT (%) | 0.91% | 1.09% |
|  |  | MCV (fL) | 0.60% | 0.42% |
|  |  | MCH (pg) | 0.94% | 0.94% |
|  |  | MCHC (g/L) | 0.92% | 1.28% |
|  |  | PLT (10^9^/L) | 4.96% | 4.99% |

**TABLE S5 Coefficients of variation (CV) under the manual mode and the autoloader mode of the Mindray BC-6800 Plus by analazing HQCM**

| HQCM | Concentrations | Parameters | Manual Mode CV | Autoloader Mode CV |
| --- | --- | --- | --- | --- |
| Bioyuan | Low | WBC (10^9^/L) | 2.83% | 3.15% |
|  |  | RBC (10^12^/L) | 1.17% | 0.76% |
|  |  | HGB (g/L) | 1.35% | 1.37% |
|  |  | HCT (%) | 0.85% | 1.31% |
|  |  | MCV (fL) | 0.34% | 0.45% |
|  |  | MCH (pg) | 1.07% | 1.06% |
|  |  | MCHC (g/L) | 0.95% | 0.94% |
|  |  | PLT (10^9^/L) | 4.60% | 3.37% |
|  | Medium | WBC (10^9^/L) | 1.54% | 2.02% |
|  |  | RBC (10^12^/L) | 0.87% | 0.64% |
|  |  | HGB (g/L) | 0.72% | 0.72% |
|  |  | HCT (%) | 0.89% | 0.90% |
|  |  | MCV (fL) | 0.52% | 0.52% |
|  |  | MCH (pg) | 0.68% | 0.66% |
|  |  | MCHC (g/L) | 0.65% | 0.64% |
|  |  | PLT (10^9^/L) | 2.94% | 2.82% |
|  | High | WBC (10^9^/L) | 1.65% | 1.16% |
|  |  | RBC (10^12^/L) | 0.71% | 0.91% |
|  |  | HGB (g/L) | 0.60% | 0.61% |
|  |  | HCT (%) | 1.07% | 1.10% |
|  |  | MCV (fL) | 0.61% | 0.61% |
|  |  | MCH (pg) | 0.67% | 0.68% |
|  |  | MCHC (g/L) | 0.66% | 0.67% |
|  |  | PLT (10^9^/L) | 2.06% | 2.18% |
| XC | Low | WBC (10^9^/L) | 2.79% | 3.23% |
|  |  | RBC (10^12^/L) | 1.15% | 0.78% |
|  |  | HGB (g/L) | 0.53% | 1.01% |
|  |  | HCT (%) | 0.97% | 0.92% |
|  |  | MCV (fL) | 0.44% | 0.44% |
|  |  | MCH (pg) | 0.84% | 0.88% |
|  |  | MCHC (g/L) | 0.91% | 1.41% |
|  |  | PLT (10^9^/L) | 4.48% | 4.98% |
|  | Medium | WBC (10^9^/L) | 1.76% | 2.13% |
|  |  | RBC (10^12^/L) | 0.69% | 0.71% |
|  |  | HGB (g/L) | 0.61% | 0.36% |
|  |  | HCT (%) | 0.77% | 0.66% |
|  |  | MCV (fL) | 0.35% | 0.40% |
|  |  | MCH (pg) | 0.52% | 0.71% |
|  |  | MCHC (g/L) | 0.70% | 0.72% |
|  |  | PLT (10^9^/L) | 4.80% | 4.58% |
|  | High | WBC (10^9^/L) | 1.64% | 1.52% |
|  |  | RBC (10^12^/L) | 0.68% | 0.69% |
|  |  | HGB (g/L) | 0.23% | 0.35% |
|  |  | HCT (%) | 0.99% | 1.12% |
|  |  | MCV (fL) | 0.62% | 0.65% |
|  |  | MCH (pg) | 0.92% | 0.83% |
|  |  | MCHC (g/L) | 1.16% | 1.23% |
|  |  | PLT (10^9^/L) | 3.81% | 3.59% |

**TABLE S6 IQC data obtained using the instrument manufacturer-provided QC materials**

| Concentrations | Parameters | Sysmex XN-1000 | | | Mindary BC-6800Plus | | | Performance Standard of Precision |
| --- | --- | --- | --- | --- | --- | --- | --- | --- |
|  |  | Mean | SD | CV | Mean | SD | CV |  |
| L | WBC (10^9^/L) | 3.08 | 0.07 | 2.2% | 3.85 | 0.11 | 3.0% | 6.0% |
|  | RBC (10^12^/L) | 2.33 | 0.03 | 1.2% | 2.34 | 0.02 | 1.0% | 3.0% |
|  | HGB (g/L) | 56.95 | 0.38 | 0.7% | 59.81 | 0.59 | 1.0% | 2.5% |
|  | HCT (%) | 17.08 | 0.30 | 1.8% | 19.90 | 0.21 | 1.1% | 5.0% |
|  | MCV (fL) | 73.15 | 0.60 | 0.8% | 85.08 | 0.56 | 0.7% | 3.0% |
|  | MCH (pg) | 24.43 | 0.28 | 1.2% | 25.55 | 0.32 | 1.2% | 3.0% |
|  | MCHC (g/L) | 333.35 | 5.42 | 1.6% | 300.81 | 4.38 | 1.5% | 4.0% |
|  | PLT (10^9^/L) | 90.80 | 3.99 | 4.4% | 59.45 | 4.79 | 8.1% | 10.0% |
| M | WBC (10^9^/L) | 6.77 | 0.09 | 1.4% | 7.47 | 0.19 | 2.5% | 4.5% |
|  | RBC (10^12^/L) | 4.25 | 0.04 | 1.1% | 4.27 | 0.03 | 0.7% | 2.5% |
|  | HGB (g/L) | 110.40 | 0.97 | 0.9% | 121.57 | 0.73 | 0.6% | 2.0% |
|  | HCT (%) | 33.12 | 0.48 | 1.4% | 39.37 | 0.42 | 1.1% | 4.0% |
|  | MCV (fL) | 77.95 | 0.42 | 0.5% | 92.30 | 0.61 | 0.7% | 2.5% |
|  | MCH (pg) | 25.99 | 0.33 | 1.3% | 28.48 | 0.21 | 0.7% | 2.5% |
|  | MCHC (g/L) | 333.05 | 4.99 | 1.5% | 308.86 | 2.29 | 0.7% | 3.0% |
|  | PLT (10^9^/L) | 248.45 | 4.35 | 1.8% | 236.62 | 10.82 | 4.6% | 8.0% |
| H | WBC (10^9^/L) | 16.67 | 0.17 | 1.0% | 20.94 | 0.37 | 1.8% | 4.5% |
|  | RBC (10^12^/L) | 5.03 | 0.04 | 0.9% | 5.08 | 0.04 | 0.7% | 2.5% |
|  | HGB (g/L) | 146.25 | 0.99 | 0.7% | 154.52 | 0.96 | 0.6% | 2.0% |
|  | HCT (%) | 42.57 | 0.51 | 1.2% | 50.67 | 0.50 | 1.0% | 4.0% |
|  | MCV (fL) | 84.66 | 0.50 | 0.6% | 99.81 | 0.64 | 0.6% | 2.5% |
|  | MCH (pg) | 29.09 | 0.36 | 1.2% | 30.45 | 0.25 | 0.8% | 2.5% |
|  | MCHC (g/L) | 343.65 | 4.64 | 1.4% | 305.10 | 2.35 | 0.8% | 3.0% |
|  | PLT (10^9^/L) | 583.40 | 11.91 | 2.0% | 443.62 | 14.60 | 3.3% | 8.0% |
